# Haematological indices of sickle cell patients with chronic leg ulcers on compression therapy

**DOI:** 10.4102/ajlm.v9i1.1037

**Published:** 2020-12-21

**Authors:** Oluwatoyin A. Babalola, Ayodele Ogunkeyede, Abayomi B. Odetunde, Foluke Fasola, Anthony A. Oni, Chinedum P. Babalola, Adeyinka G. Falusi

**Affiliations:** 1 Institute for Advanced Medical Research and Training, College of Medicine, University of Ibadan, Ibadan, Nigeria; 2 Department of Surgery, University of Ilorin Teaching Hospital, Ilorin, Kwara State, Nigeria; 3 Department of Haematology, College of Medicine, University of Ibadan, Ibadan, Nigeria; 4 Department of Medical Microbiology, College of Medicine, University of Ibadan, Ibadan, Nigeria; 5 Sickle Cell Hope Alive Foundation (SCHAF), Nigeria

**Keywords:** chronic leg ulcer, sickle cell anaemia, wound healing, haematological parameters, compression therapy

## Abstract

**Background:**

Recurrent chronic leg ulcers and its are morbidities associated with sickle cell anaemia (SCA). Compression therapy increases the rate of healing of these ulcers and also decreases the rate of recurrence.

**Objective:**

This study evaluated the haematological parameters of patients with SCA and chronic leg ulcers placed on high compression bandaging to provide data for improved ulcer management and prevention.

**Methods:**

Eighteen patients with SCA and chronic leg ulcers were recruited for treatment by compression therapy in Ibadan, Nigeria, from March to June 2015. Eighteen SCA patients with no history of chronic leg ulcers were age and sex matched and recruited as controls. Blood samples, wound biopsies and swabs were collected at different time points for full blood count, microbiology, culture and antimicrobial susceptibility tests. Haemoglobin variants were quantified by high performance liquid chromatography. Fasting blood sugar was tested for leg ulcer patients to determine diabetic status.

**Results:**

Ulcers ranged from 0.5 cm^2^ to 416 cm^2^ (median: 38.4 cm^2^). Post-intervention ulcer size ranged from 0.0 cm^2^ to 272 cm^2^ (median: 18.6 cm^2^, *p* < 0.001); four ulcers completely healed. Compared to the control group, haematological indices at commencement of treatment were more severe in leg ulcer patients (*p* = 0.02). No patients with chronic leg ulcer was diabetic. Microorganisms isolated from the leg ulcers include *Pseudomonas aeruginosa, Staphylococcus aureus, Proteus* sp., *Escherichia coli* and *Klebsiella oxytoca*.

**Conclusion:**

Measures to improve haematological parameters during leg ulcer treatment in SCA patients should be taken to aid wound healing.

## Introduction

Chronic leg ulcer is one of the morbidities associated with sickle cell anaemia (SCA). Chronic leg ulcer is a wound on the skin that occurs between the knee and the foot, showing no signs of healing after 3 months of proper treatment.^1^ In SCA patients, the polymerisation of haemoglobin S (HbS) leads to the formation of sickled red blood cells (RBCs) which become trapped in the microcirculatory system. This leads to microvascular vaso-occlusion causing tissue ischaemia and associated pain. Occlusion of the veins in the legs leads to venous hypertension, which may cause varicose eczema, deposition of scar tissue and iron pigments in the skin, and oedema in the lower leg. This may result in the breakdown of the skin or delay healing in the case of a leg injury, leading to chronicity.^2^ Any leg ulcer in the presence of a venous disorder is described as a venous ulcer. Other considerations for venous ulcer diagnosis are an ankle brachial pressure index greater than 0.8 with absence of features of rheumatoid vasculitis, malignancies and diabetic mellitus. The gold standard in the management of chronic venous leg ulcers is the four-layered compression therapy bandaging system.

Sickle cell associated ulcers (SCUs) can be precipitated by many factors such as trauma, infection, severe anaemia and usually occur with venous stasis disease, which requires compression therapy.^2^ The microorganisms invading the tissues of the skin contribute to the delayed healing observed in chronic leg ulcers.^3^ Bacteria produce and also stimulate the production of substances (from activated neutrophils) that are detrimental to the body’s wound healing processes.^4,5^ These substances include proteases such as metalloproteases, elastases and collagenases that destroy the building blocks needed for normal wound healing. In this proteolytic environment, bacteria can continue to multiply and use up local resources such as oxygen and nutrients that are also needed for the wound healing.^4^ Diabetes mellitus increases the risk for the development of infection and impaired wound healing in individuals due to a decreased cell and growth factor response.^6^ The effect of diabetes mellitus in SCUs is yet to be assessed.

Ulcer incidence in sickle cell disease (SCD) patients in the United States ranges from 2.5% to 25% and 75% in Jamaica.^7,8^ In Nigeria and Ghana, it is about 10%,^9,10^ and the rate of recurrence of ulcers is high (> 70%).^9,11,12^ Chronic leg ulcer thus contributes to recurrent and prolonged misery in these patients. Compression therapy increases the rate of healing of SCUs and also reduces the rate of recurrence.^7,8,9^ There is inadequate education on the prevention and management of leg ulcers among people living with SCD and their caregivers. The therapeutic strategy for SCUs is complex and multidisciplinary efforts to offer holistic care is poor. This study is therefore designed to address the paucity of information about the laboratory markers of SCUs among people living with SCD and also serves as a basis for health promotion interventions for the prevention of SCUs and improvement in clinical interventions in Nigeria and other African countries.

## Methods

### Ethical considerations

This study was approved by the University of Ibadan/University College Hospital Joint Ethics Review Committee, College of Medicine, University of Ibadan; protocol identification number UI/EC/15/0133. Sickle cell disease patients in need of leg ulcer treatment at the haematological clinics of the University College Hospital and Ring Road State Hospital, Ibadan, voluntarily participated in this study. Oral informed consent was obtained from each of the participants using a format explaining the details of the procedure, possible complications that may arise using the compression therapy technique for wound care and the importance of the biopsies and periodic blood sample collection. A code number was assigned to each patient and their samples to protect participants’ privacy.

### Study design

In a pilot hospital-based study, 18 SCA patients with chronic leg ulcers were recruited from haematology clinics at University College Hospital and Ring Road State Hospital, Ibadan, for treatment by compression therapy (first use case in Nigeria) for a period of 3 months (13 weeks) between March 2015 and June 2015. There were 6 men and 12 women with ages ranging from 19 to 44 years. Eighteen age and sex matched SCA patients with no history of chronic leg ulcers were recruited as controls. Sickle cell anaemia patients below 18 years as well as adult SCA patients attending the named facilities who did not give informed consent were excluded from this study. Those with chronic leg ulcers with an ankle brachial pressure index less than 0.8 were also excluded. A pre-tested semi-structured questionnaire was administered to each of the patients for a history of leg ulcer, family history and socio-demographic details.

A four-layer high compression bandaging system described previously.^13^ was used in this study. Dressing was done once a week for 13 weeks.

### Sample collection and processing

Blood samples were collected before commencement of treatment (T_0_) from both groups. For the leg ulcer group, blood samples, wound swabs and tissue biopsies (in normal saline and in peptone) were also collected on the 5th week (T_1_) and 10th week (T_2_) of treatment. Microscopy, culture and sensitivity tests were carried out on the wound biopsies and swabs. Full blood count was carried out using a Swelab Haematology Analyser (Boule Diagnostics, Stockholm, Sweden) and the level of haemoglobin variants was determined by high performance liquid chromatography using the BioRad Variant II Hemoglobin Testing System (Clinical Diagnostics, Hercules, California, United States). The patients were prescribed antibiotics based on the result of antibiotic sensitivity tests. A fasting blood sugar test was performed for the leg ulcer patients to determine their diabetic status.

### Statistical analysis

Statistical analyses were done using IBM Statistical Package for Social Sciences version 23 (IBM Corporation, Armonk, New York, United States). The differences in haematological parameters between the control and test groups were determined by the student’s *t*-test. The paired *t*-test was used to evaluate changes in haematological parameters within chronic leg ulcer patients during the therapy. Spearman’s rank correlation was used to determine relationships between different haematological parameters and between haematological indices and size of ulcers at T_0_. The level of statistical significance was set at *p* < 0.05.

## Results

### Description of patients, ulcers and healing rate

Eighteen patients with leg-ulcers and a similar number of ulcer-free controls were included in the study. There were 25 uclers in all as seven patients had bilateral ulcers. The ulcers ranged from 0.5 cm^2^ to 416 cm^2^ with a median ulcer size of 38.4 cm^2^. The ages of the ulcers were 1–22 years (with recurrence). Within the 3 months of compression therapy, four of the patients had complete close-up of the ulcer wound, 16 of them had more than a 50% healing rate (the percentage of the size of wound closure to the initial wound size) and the remaining five had a healing rate of 35% – 50%. The post-intervention median ulcer size ranged from 0.0 cm^2^ to 272 cm^2^ with a median of 18.6 cm^2^ (*p* < 0.001). The full details of patients, ulcers and healing rates have been described elsewhere.^12^

### Full blood count analysis

Haematocrit (HCT) at commencement of treatment was lower in the leg ulcer patients compared to the control group (19.11% vs 26.17%, *p* < 0.001). Mean corpuscular volume (95.33 femtolitre vs 87.73 femtolitre, *p* = 0.02), mean corpuscular haemoglobin (MCH) (30.82 picogram vs 28.46 picogram, *p* = 0.05), red cell distribution width (27.78% vs 22.68%, *p* = 0.004), white blood cell count (WBC) (13.90 vs 8.70, *p* < 0.001) and platelet count (PLT) (487.42 vs 299.08, *p* < 0.001) were higher in the leg ulcer patients (Table 1). In the sickle cell patients, HCT at T_2_ was significantly higher than at T_0_ (*p* = 0.04). There was an improvement in most of the haematological indices from T_0_ to T_2_, but that between T_0_ and T_1_ was more profound than T_1_ to T_2_ (Table 1). Elevated WBC was more prevalent in leg ulcer patients (66.7%) at T_0_ compared to the control group (11.1%) *p* < 0.001.

**TABLE 1:** Mean values for full blood count parameters across the different time periods in adult sickle cell anaemia patients with (cases) and without (controls) chronic leg ulcers at the University College Hospital and Ring Road State Hospital, Ibadan, Nigeria, March–June 2015.

Time period	HGB (g/dL)	RBC (10^12^/L)	HCT (%)	MCV (fL)	RDW %	MCH (pg)	MCHC (g/dL)	WBC (10^3^/µL)	LYMF (%)	MID %	GRAN (%)	PLT (10^3^/*µ*L)	MPV (*µ*m^3^)	PCT %	PDW (*µ*m^3^)
T_0_	6.17 ± 1.37	2.05 ± 0.67	19.11 ± 4.44	95.33 ± 10.91	27.78 ± 7.54	30.82 ± 3.85	32.59 ± 1.36	13.90 ± 4.9	38.03 ± 10.35	7.07 ± 2.17	54.88 ± 12.01	487.42 ± 193.92	8.75 ± 0.65	0.42 ± 0.17	10.99 ± 1.48
T_1_	6.54 ± 1.31	2.16 ± 0.67	19.79 ± 4.35	93.85 ± 10.54	25.62 ± 3.03	31.26 ± 3.96	33.26 ± 1.41	13.1 ± 2.90	39.58 ± 8.89	5.21± 1.43	55.02 ± 9.30	408.71 ± 137.04	8.69 ± 0.54	0.36 ± 0.11	11.32 ± 0.75
T_2_	6.62 ± 1.28	2.24 ± 0.69	20.58 ± 4.29	94.99 ± 11.22	25.82 ± 3.75	30.68 ± 4.11	32.22± 0.99	11.88 ± 4.14	39.36 ± 10.0	7.62 ± 1.87	50.78 ± 15.37	495.62 ± 191.50	9.58 ± 1.25	0.47 ± 0.17	12.16 ± 1.64
Control group	8.43 ± 1.18	3.03 ± 0.6	26.17 ± 3.98	87.73 ± 11. 21	22.68 ± 2.73	28.46 ± 4.45	32.3 ± 1. 49	8.70 ± 1.92	45.74 ± 12.64	8.14 ± 3.8	46.97 ± 13.07	299.08 ± 109.03	8.68 ± 0.93	0. 26 ± 0.1	11.30 ± 1.21
*p*-value (diff. T_0_ & control)	< 0.001	< 0.001	< 0.001	0.02	0.004	0.05	0.27	< 0.001	0.03	0.15	0.03	< 0.001	0.42	< 0.001	0.25

HGB, haemoglobin concentration; RBC, red blood cell count; HCT, haematocrit; MCV, mean corpuscular volume; RDW, red cell distribution width; MCH, mean corpuscular haemoglobin; MCHC, mean corpuscular haemoglobin concentration; WBC, white blood cell count; LYMF, lymphocyte count; GRAN, granulocyte count; MID, other white blood cells that are not lymphocytes nor granulocytes; PLT, platelet count; MPV, mean platelet volume; PCT, volume of platelets in blood; PDW, platelet distribution width.

T_0_, Before commencement of treatment; T_1_, 5^th^ week of treatment; T_2_, 10^th^ week of treatment.

**FIGURE 1:**
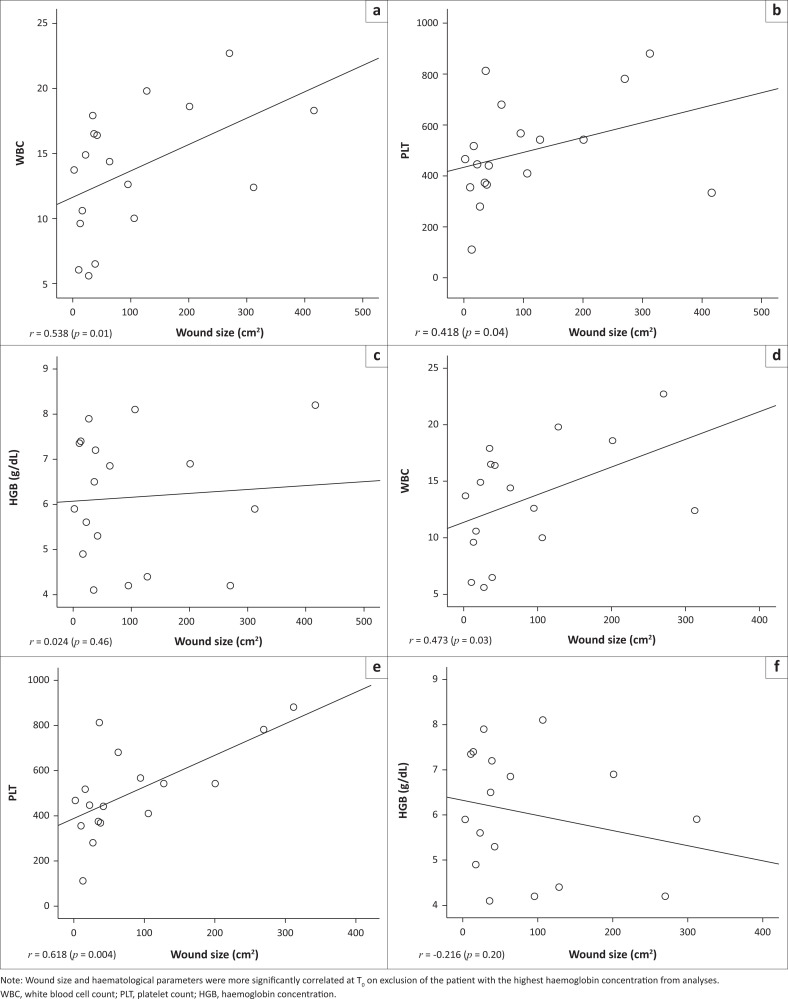
Correlation between wound size and some haematological parameters at T_0_ in adult sickle cell anaemia patients with chronic leg ulcers at the University College Hospital and Ring Road State Hospital, Ibadan, Nigeria, March–June 2015. Correlation between wound size (cm^2^) and (a) Wound size versus white blood cell count (10^3^/*µ*L), (b) Wound size versus platelet count (10^3^/*µ*L) and (c) Wound size versus haemoglobin concentration (g/dL) in all the leg ulcer patients. Correlation between wound size (cm^2^) and (d) Wound size versus white blood cell count (10^3^/*µ*L), (e) Wound size versus platelet count (10^3^/*µ*L) and (f) Wound size versus haemoglobin concentration (g/dL) excluding one leg ulcer patient with the highest haemoglobin concentration and biggest wound size.

There was a positive correlation between wound size and WBC (0.538, *p* = 0.01, Figure 1a) and also between wound size and PLT (0.418, *p* = 0.04, Figure 1b). There was a poor correlation between wound size and haemoglobin (HGB) (0.024, *p* = 0.46, Figure 1c). The leg ulcer patient with the highest HGB (8.2 g/dL) in this study test group had the biggest ulcer (416 cm^2^). On exclusion of this patient, the positive correlation between wound size and WBC became *r* = 0.473, *p* = 0.03 (Figure 1d) and that between wound size and PLT became more significant at *r* = 0.618, *p* = 0.004 (Figure 1e). However, that between wound size and HGB became *r* = -0.216, *p* = 0.20 (Figure 1f).

In the patients with chronic leg ulcers, there was a positive correlation between WBC and PLT and negative correlations between WBC and HGB as well as between HGB and PLT with and without exclusion of the patient with the biggest ulcer at T_0_. There was no correlation between any of these three parameters in the patients who had not experienced a chronic leg ulcer in their lifetime (i.e. the control group) (Table 2).

**TABLE 2:** Correlation between white blood cell count, platelet count and haemoglobin concentration at T_0_ in adult sickle cell anaemia patients with (cases) and without (controls) chronic leg ulcers at the University College Hospital and Ring Road State Hospital, Ibadan, Nigeria, March 2015 – June 2015.

Haematological parameters	Cases (*n* = 18)		Cases (*n* = 17)		Controls (*n* = 18)	
	PLT	HGB	PLT	HGB	PLT	HGB
**WBC**						
*r* value	*r* = 0.474	*r* = −0.480	*r* = 0.596	*r* = −0.671	*r* = 0.280	*r* = −0.30
*p* value	*p* = 0.02	*p* = 0.02	*p* = 0.006	*p* = 0.002	*p* = 0.13	*p* = 0.45
**HGB**						
*r* value	*r* = −0.553	-	*r* = −0.481	-	*r* = 0.230	-
*p* value	*p* = 0.009	-	*p* = 0.03	-	*p* = 0.18	-

PLT, platelet count; HGB, haemoglobin concentration; WBC, white blood cell count.

### High performance liquid chromatography analysis

The concentration of HbS was higher in the leg ulcer group (86.25% vs 82.87%, *p* = 0.03). The difference in the mean foetal haemoglobin (HbF) level between the leg ulcer group (6.01% ± 3.10%) and control group (7.49% ± 5.89%) was not statistically significant (*p* = 0.19) but four of the control patients and only one of the leg ulcer patients had a HbF level greater than 10%.

### Microbial culture test

Bacteria isolated from the wound swabs and biopsies include *Pseudomonas aeruginosa, Staphylococcus aureus, Proteus mirabilis, Escherichia coli* and *Klebsiella oxytoca*, with *Pseudomonas aeruginosa* being the most prevalent (55% – 70%) across the different culture tests.

### Fasting blood sugar test

None of the leg ulcer patients was diabetic. Results of the fasting blood sugar test for all the participants in the test group ranged from 64 mg/dL to 82 mg/dL.

## Discussion

This study was carried out to evaluate the haematological parameters in patients with SCA and chronic leg ulcers, and also to assess the haematological changes during healing of these ulcers while being managed with high compression bandaging, an as yet unemployed method of chronic leg ulcer treatment in Nigeria. There were marked differences in some haematological parameters between SCA patients with and without chronic leg ulcers and haematological parameters improved with healing of the ulcers. Monitoring of haematological parameters for improvement during treatment of chronic leg ulcers and after wound closure in SCA patients will aid healing and might also reduce the rate of recurrence.

The mean HGB and HCT of the case patients (those with chronic leg ulcers) at T_0_ were significantly lower than those of the control patients (those who have never had a chronic leg ulcer). A high rate of haemolysis is one of the major causes of a reduced steady state haemoglobin in sickle cell patients. Sickle cell anaemia patients with a lower steady state haemoglobin concentration are at a higher risk of developing chronic leg ulcer.^14,15^ According to Ladizinski et al.,^16^ the inability of a wound to heal could be a result of an inadequate supply of blood or a very low haemoglobin level (< 8 g/dL). One of the causes of SCUs is lack of oxygen which may be attributable to overall anaemia and reduced oxygen-carrying capacity of sickled haemoglobin.^8^ The addition of recombinant human erythropoietin (a glycoprotein hormone that stimulates red blood cell production) subcutaneously at weekly intervals resulted in a rapid and complete healing of a chronic leg ulcer.^17^ Hence, treatment of chronic leg ulcers should aim at anaemia correction. However, this should be done with caution and within a threshold that will be beneficial to the patient as a much higher HCT may also lead to hyperviscosity and increase the risk of vaso-occlusion.

A wider red cell distribution width as compared to the control group, with a mean corpuscular volume within normal haematological range, in the test patients alludes to anaemia caused by bleeding from regular treatments, folate deficiency or vitamin B12 deficiency. There was a slight improvement in the HCT level from T_0_ to T_2_ mainly due to constant education of the patients on the need to improve the quality of their diet and better compliance with routine haematinics.

Nolan et al.^14^ and Hassan et al.^18^ found that patients with leg ulcers had higher WBCs as is the case in this study. The majority of the patients with leg ulcers had elevated WBC counts at the beginning of the study. Elevated WBC, which is due to recurrent inflammation and vasculopathy, is one of the hallmarks of disease severity.^19,20,21^ Patients with elevated WBC may be more susceptible to developing chronic leg ulcer because leukocytes stick to other blood cells and the endothelium due to an upregulation of adhesion molecules, thereby promoting vaso-occlusion.^22^ Vaso-occlusion of the veins in the leg may cause tissue ischaemia with a resultant breakdown of the skin or delayed healing of wounds, leading to chronicity.^2^ Higher WBC may also be due to microbial colonisation or infection of the ulcers. A lower WBC count was observed at T_2_ and this may be due in part to the use of antibiotics prescribed (based on the result of an antibiotic sensitivity test) a week before T_1_ sampling. Infections that are not resolved may lead to the non-healing of wounds.^16^ There is a the need to control infections to promote wound closure and healing. In this study, wound size was positively correlated with WBC. Therefore, efforts to reduce elevated WBC in leg ulcer treatment may aid wound closure.

There was a significantly higher number of platelets in the leg ulcer patients than in the control group of this study. In Jamaica, Cumming et al.^23^ found that leg ulceration was associated with higher PLT. Hypercoagulability can trigger leg ulcer formation as it may cause ischaemia to the skin, resulting in friability and ulceration.^24^ SCUs are characterised partly by an increase in clotting ability as a result of increased PLT, hypercoagulability and a measured increase in clotting factors at the wound itself.^7^ In this study, PLT was also positively correlated with wound size. Wirth et al.^25^ asserted that an efficient decrease in PLT may lead to healing of skin ulcers.

There was a negative correlation between HGB and WBC as well as between HGB and PLT. This means a higher rate of haemolysis may result in elevated WBC and PLT counts. The observation that these three parameters were not significantly correlated in patients without chronic leg ulcers lends credence to the notion that lower steady state HGB and elevated WBC and PLT counts may predispose sickle cell patients to chronic leg ulcers.

Koshy et al.^15^ noted that there was a lower incidence of leg ulcers in SCD patients with a HbF concentration higher than 10%. In Jamaica, Cumming et al.^23^ also found that ulceration was negatively associated with foetal haemoglobin levels. In this study however, the difference in HbF levels between the two study groups was not statistically significant, probably due to the small sample size. The presence of HbF may help to reduce the rate of haemolysis by reducing sickling potential. Having HbF within the cell reduces its ability to polymerise due to the formation, in HbS and HbF mixtures, of asymmetrical hybrid tetramers of the type α_2_β^S^γ. The contact sites of Hb β^S^ for polymerisation is abolished, and hence prevents sickling^26^ and this may also reduce subsequent inflammatory reactions. However, elevated HbF levels may not always be protective as the pancellular distribution, rather than the concentration of HbF in the haemolysate is more effective in inhibiting polymerisation.^27^

The level of HbS was lower in the control patients. A lower HbS level is usually due to higher HbF levels or the presence of alpha or beta thalassemia which was not assayed in this study. Reduced HbS concentration leads to a lower rate of sickling and, consequently, a reduced rate of haemolysis. Haemolysis of sickled RBCs leads to the release of activated molecules (plasma haemoglobin) which react with nitric oxide to form inert nitrate. Nitric oxide maintains vascular function and normal blood flow^28,29^ and has also been shown to be vital to cutaneous wound healing.^30,31^ Nitric oxide suppresses aggregation of platelets, secretion of procoagulant proteins and the expression of cell adhesion molecules on endothelial cells, thereby promoting blood flow.^28^ Hence, improvement in leg ulcer management may be achieved by the introduction of agents that have the capacity to reduce sickle erythrocyte density as well as those that can make nitric oxide more available in tissues.

None of the chronic leg ulcer patients in this study was diabetic. Diabetes is quite rare in sickle cell patients^6,32,33^ and does not have anything to do with the non-healing ulcers of patients in this study. Venous incompetence leads to reduced blood flow, and may cause increased blood vessel permeability with the attendant leaking of fluid and proteins into tissue spaces, a condition known as oedema.^34^ Localised oedema traps leukocytes in the tissues, and oxygen-free radicals and harmful substances released by the leukocytes are capable of causing tissue damage, which may eventually lead to the development of ulceration. Blood clotting factors are also triggered in reduced blood flow.^35,36^ According to Clare et al.,^37^ ‘hypoxia-induced sickling, the rheological effects of white cell counts, and activation of components of the coagulation system may all contribute’ to venous incompetence. This explains why reduced HCT, elevated PLT and WBC may increase the risk of leg ulceration in individuals with SCD.

Compression therapy aids healing of leg ulcers by increasing venous blood flow,^38^ thereby making more readily available the supply of adequate nutrients and other factors necessary for wound healing. On the other hand, the risk of blood clot formation and oedema is reduced, thereby promoting healing. However, there may be a recurrence of ulcers if haematological conditions are not monitored and addressed. This was the case for 3 of the previously healed participants in this study. Management and prevention of chronic leg ulcer in SCD patients should address underlying haematological indices as no one method of dressing may totally prevent recurrence.^39,40^ The wearing of compression stockings after wound healing may help to maintain normal venous pressure and prevent recurrence due to skin tissue breakdown.^41,42^

### Recommendations

Chronic leg ulcer is multifactorial and hence, in order to make the correct diagnosis and give the most effective treatment, health practitioners should adopt a multidisciplinary approach to a systematic patient evaluation to determine the ulcer pathogenesis.^34^ Laboratory investigations to monitor haematological indices and to reveal any underlying infection should be incorporated in the treatment and management modalities of chronic leg ulcer in Nigeria. Agents that will reduce the rate of sickle red blood cell destruction and cell adherence to endothelial venules should be explored by health practitioners in the prevention of the formation and recurrence of ulcers and also in its management. Several chemical agents have been suggested and some are under clinical trials.^43^ Some investigators^44,45^ have also shown that ascorbic acid favours nitric oxide production in endothelial cells by stabilising nitric oxide synthase cofactor tetrahydrobiopterin. It is therefore suggested that the inclusion of vitamin C at recommended doses be explored in the daily routine drugs for individuals living with SCD for the prevention and management of chronic leg ulcers and other haemolysis-related sickle cell phenotypes.

### Limitations

One limitation of this study is the small sample size. The high cost of compression therapy was the major determinant of the sample size. Laboratory markers such as elevated serum lactose dehydrogenase, reticulocyte count, bilirubin level and some genetic factors which were not assayed in this study have also been associated with a risk of chronic leg ulcers in patients with SCD,^7,14^ Assaying for all these factors in a larger cohort of patients may help to identify other laboratory markers of chronic leg ulcer susceptibility.

### Conclusion

Sickle cell patients with a lower level of HGB and elevated WBC and PLT counts at steady state may be at a higher risk of non-healing leg ulcers. Most of the haematological indices improved with healing of the leg ulcers placed on high compression bandaging. An interdisciplinary approach to provide a favourable microenvironment for healing of ulcers, and the monitoring of haematological parameters for the prevention of recurrence, is recommended.
